# Sparse testing using genomic prediction improves selection for breeding targets in elite spring wheat

**DOI:** 10.1007/s00122-022-04085-0

**Published:** 2022-03-28

**Authors:** Sikiru Adeniyi Atanda, Velu Govindan, Ravi Singh, Kelly R. Robbins, Jose Crossa, Alison R. Bentley

**Affiliations:** 1 International Maize and Wheat Improvement Center (CIMMYT), Texcoco, Mexico; 2 Section of Plant Breeding and Genetics, School of Integrative Plant Sciences, Cornell University, Ithaca, NY USA

## Abstract

**Key message:**

Sparse testing using genomic prediction can be efficiently used to increase the number of testing environments while maintaining selection intensity in the early yield testing stage without increasing the breeding budget.

**Abstract:**

Sparse testing using genomic prediction enables expanded use of selection environments in early-stage yield testing without increasing phenotyping cost. We evaluated different sparse testing strategies in the yield testing stage of a CIMMYT spring wheat breeding pipeline characterized by multiple populations each with small family sizes of 1–9 individuals. Our results indicated that a substantial overlap between lines across environments should be used to achieve optimal prediction accuracy. As sparse testing leverages information generated within and across environments, the genetic correlations between environments and genomic relationships of lines across environments were the main drivers of prediction accuracy in multi-environment yield trials. Including information from previous evaluation years did not consistently improve the prediction performance. Genomic best linear unbiased prediction was found to be the best predictor of true breeding value, and therefore, we propose that it should be used as a selection decision metric in the early yield testing stages. We also propose it as a proxy for assessing prediction performance to mirror breeder’s advancement decisions in a breeding program so that it can be readily applied for advancement decisions by breeding programs.

**Supplementary Information:**

The online version contains supplementary material available at 10.1007/s00122-022-04085-0.

## Introduction

Genomic prediction (GP) is a statistical method to predict the genetic potential of unobserved lines based on genomic information. It has been identified as a viable tool to accelerate genetic gain and to reduce phenotyping costs in plant breeding programs, particularly as genotyping costs become cheaper than phenotyping costs (Crossa et al. 2010; Juliana et al. 2019; Santantonio et al. 2020; Atanda et al. 2021a). GP is a flexible approach that can be implemented at different stages in a breeding program and for different purposes, depending on the objectives and overall breeding strategy.

The CIMMYT global spring wheat breeding program uses two yield testing stages to identify parents for the next breeding cycle and promising candidates to advance based on high and stable yield across managed selection environments (SEs) (Suppl. Figure 1). These candidates are then tested internationally through collaborative trials with partners selecting elite lines for use as parents in national breeding programs and/or for variety release. The SEs are defined by varying sowing time and management conditions in a single location (Ciudad Obregon, Mexico). Although the SEs were defined within a single location, they are constructed to predict the performance in global target populations of environments (Crespo-Herrera et al. 2021). In the initial yield testing stage (denoted PYT, or stage 1), lines with desirable agronomic and grain traits, and resistance to diseases, especially rusts, are evaluated for yield potential in an optimal five irrigations bed planting environment to discard low yielding lines while maintaining a range of maturity. Accurately capturing genotype x environment (GxE) interaction is critical in identifying promising lines with the greatest potential to perform in international trials (Falconer and Mackay 1996; Cooper et al. 1995; Mohammadi and Amri 2011). Therefore, selected lines from stage 1 are further evaluated in a subsequent Elite Yield Trial (denoted EYT, or stage 2) in six SEs. This two-stage process of identifying superior performing lines is time consuming and costly, requiring two consecutive cycles of yield testing. Therefore, a key objective of the CIMMYT spring wheat program is shortening the selection cycle by advancing lines directly to multi-environment trials in year 1 using GP to discard lines with low genomic best linear unbiased predictions (GBLUPs) for grain yield and other relevant traits (Suppl. Figure 1).

Sparse testing, in which the phenotyping of lines is split across environments, is a robust strategy to help achieve two objectives, specifically (1) increased number of lines tested across multiple, diverse environments and (2) increased number of testing environments while maintaining the same selection intensity (Burgueño et al. 2012; Jarquin et al. 2020; Atanda et al. 2021b). The latter is the proposed usage of GP in CIMMYT spring wheat breeding where phenotypic data across SEs serve as a calibration model to predict GBLUPs to make earlier selection of promising lines for international trials (Suppl. Figure 1).

The size of the full-sib family in the CIMMYT spring wheat breeding program in the early yield testing stage is relatively small. Therefore, the dataset used in this study, individuals within a family were likely to be absent across environments (contrary to Atanda et al. (2021b)), and the size of the calibration set is relatively small in each environment. Both factors are likely to influence the prediction accuracy when applying sparse testing in the early yield testing stage. Good prediction accuracies have been reported within bi-parental populations and lower prediction accuracies across populations due to inconsistent quantitative trait loci (QTL)-marker linkage phase across populations (Clark et al. 2012; Lehermeier et al. 2014; Brandariz and Bernardo 2019; Atanda et al. 2021a). However, in a scenario where the prediction and calibration set are heterogeneous, the effect of changes in LD-marker phase across populations on prediction accuracy might be minimal. Consequently, we evaluated the efficiency of sparse testing with GP when the prediction and calibration set are heterogeneous using different sparse testing strategies defined by the proportion of overlapping lines across SEs to identify an optimal strategy without sacrificing selection accuracy. The sparse testing is implemented in stage 1 (where current stage 2 will become stage 1), and thus the dataset used here mimics the data architecture expected in direct stage 2 by skipping stage 1 when GBLUP will be used to select promising lines for further testing (Suppl. Figure 1). In addition, past breeding lines with genotypic and phenotypic data in relevant environments constitute a resource to increase the size of the calibration set (Mangin et al. 2019; Brandariz and Bernardo 2019; Auinger et al. 2021; Atanda et al. 2021a). Here, we also evaluate the merit of using past breeding information to increase the size of training set without increasing costs for sparse testing using GP.

In most plant breeding programs, the genetic value of genotypes is estimated through adjusted best linear unbiased estimates (BLUEs) (Falconer and Mackay 1996; Santantonio et al. 2020; Bernardo 2020; Lell et al. 2021). Theoretically, breeding value is a predictor of selection candidate potential to produce superior progenies in the next generation. However, true breeding value is unknown; thus, the efficiency of the advancement decision depends on a selection metric that is predictive of the true breeding value. In animal breeding, GBLUP estimated from phenotypic information of an individual and relatives using a marker or pedigree relationship matrix in mixed model equations is widely used as a selection metric for candidates (Zhang et al. 2011; Junjie and Shengjie 2019). Recently, adoption of GBLUP as a selection decision metric is gaining traction in plant breeding (Bernardo 2020; Lell et al. 2021), Therefore, we propose and test the use of GBLUP as an advancement decision metric for low to medium traits heritability, especially in the early yield testing stages in order to improve selection accuracy.

Prediction accuracy is often assessed as Pearson correlation between predicted GBLUP and the BLUE (Crossa et al. 2014; Zhang et al. 2019). However, this more closely reflects predictive ability rather than accuracy, and a proxy for prediction accuracy is needed to reflect the breeding program advancement decision strategy. Therefore, we assessed the prediction performance of sparse testing aided GP as the proportion of lines that overlap between select top 20% lines using the Smith Hazel selection index (Smith 1936; Hazel 1943) with GBLUP from the prediction model and GBLUP estimated from full data across the SEs.

Using breeding data from the CIMMYT spring wheat program the overall objective of this study was to test sparse testing strategies using GP via a number of approaches, namely: (1) highlighting selection accuracy using GBLUP as a selection metric for line advancement decisions in direct stage 2 skipping stage 1 testing; (2) determining the optimal sparse testing aided GP strategy in direct stage 2 trials; (3) determining the contribution of historical data to increasing the calibration set size and improving prediction accuracy of untested lines across SEs without increasing cost; and (4) determining the appropriate method for evaluating prediction accuracy to closely mirror breeder advancement decisions.

## Materials and methods

### Plant material and field evaluation

The genetic material used in this study consisted of F4:8 (stage 1) and F4:9 (stage 2) CIMMYT spring wheat breeding lines. The genetic material was classified into three datasets (denoted DS1, DS2 and DS3). DS1 consisted of 1260 stage 1 lines grouped into 45 trials, each with 28 entries and two checks, evaluated for grain yield (GY) and other agronomic traits in an optimal five irrigation bed planting SE (B5IR). Each trial was planted in an alpha-lattice incomplete block design with two replicates in the 2019–2020 crop season at Norman E. Borlaug research station, Ciudad Obregon, Sonora, Mexico (27° 29′ N, 109° 56′ W). DS2 consisted of the 280 stage 2 lines advanced from the DS1 1260 lines based on GY performance, agronomic, disease, grain zinc and processing quality traits. They were evaluated in six SEs at the same location in the 2020–2021 season. In each SE, the lines were grouped into five trials, each with 56 entries and 4 checks, and the trials were planted in an alpha-lattice incomplete block design with two replicates.

The SEs were defined by a combination of factors including planting date, irrigation, and planting condition (flat or bed) as follows:

1. Optimal planting date and five irrigations bed planting (B5IR). Approximately 500 mm of water was applied through flood irrigation. Optimal planting date implies planting during the third week of November to first week of December.
2. Optimal planting date and five irrigations flat planting (F5IR), with the same total amount of water applied as B5IR, through drip irrigation.
3. Optimal planting date and two irrigations bed planting (B2IR). Approximately 250 mm of water was applied through flood irrigation.
4. Optimal planting date and drought stress flat planting (FDRIP). Approximately 180 mm of water applied through drip irrigation.
5. Early heat stress bed planting (BEHT). Lines were planted about 3 weeks earlier (1^st^ week of November) than the optimal planting date with the aim of evaluating the lines for heat tolerance during the early growth stage. Approximately 500 mm of water was applied through flood irrigation.
6. Late heat stress bed planting (BLHT). Contrary to BEHT, lines were sown 90 days after optimal planting date to evaluate the lines for heat tolerance during the flowering and grain filling stages of the plant. Approximately 500 mm of water was applied through flood irrigation.

DS3 consisted of the stage 2 lines advanced from 2018–2019 stage 1 (data not used) and consisted of 253 lines evaluated in six SEs at the same location in the 2019–2020 season.

To account for spatial variation in the field, the trials were sown as a grid of (6, 8, 6) rows and (15, 15, 15) columns for DS1, DS2 and DS3 data, respectively.

DS2 advanced lines from DS1, and DS3 lines were genotyped using genotyping-by-sequencing (GBS) and 93,349 SNP markers were generated. After removing SNPs with more than 20% missing values and with a minor allele frequency less than 5%, 20,985 SNPs remained and were used for the analysis. Missing SNPs imputed with Beagle 5.1 (Browning et al. 2018).

### Cross-validation scheme

**Fig. 1:**
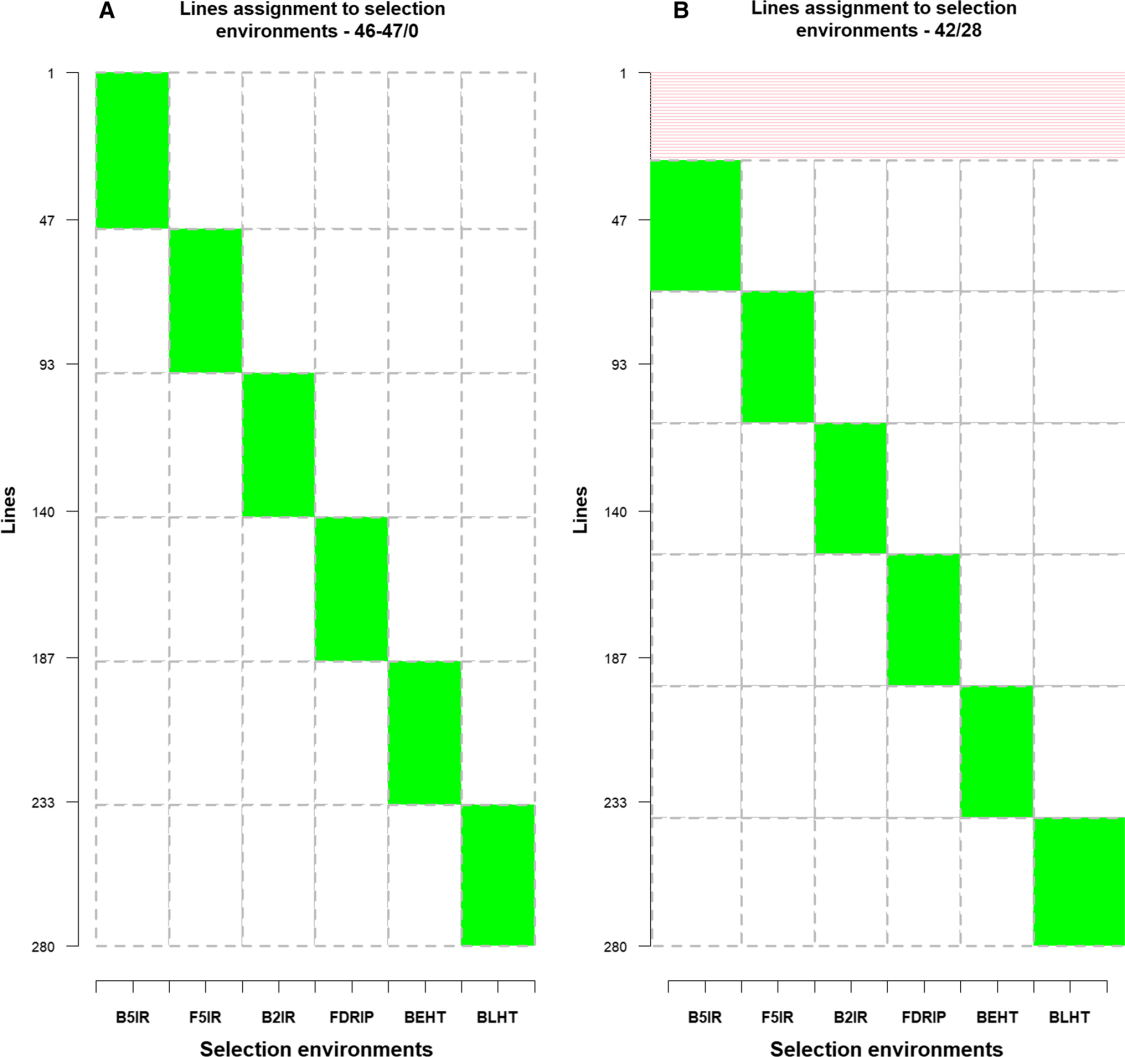
Allocation of 280 DS2 lines to the six SEs. Each column represents a discreet SE and the green sections in each column correspond to unique lines tested in each SE. A: The green sections correspond to 46 or 47 lines unique to each SE with no overlapping lines across the SEs. B: The pink section represents 28 lines (10% of DS2 lines) that overlapped across the SEs, and the green sections are the 42 DS2 lines unique to each SE

We evaluated the efficiency of two sparse testing GP scenarios based on the approach reported by Jarquin et al. (2020):

1. Phenotyping a different set of lines in each SE, i.e. lines were not repeated across SEs. In this scenario, the 280 lines in DS2 were divided into six unique sets with some SEs having 46 lines, while others had 47 lines in the calibration set (Fig. 1). Therefore, the prediction set in each SE consisted of 234 lines where 46 lines were considered tested, and 233 lines when 47 lines were considered tested in the SE. Splitting of lines across the SEs was repeated 30 times.
2. A subset of lines overlapping across the SEs to allow borrowing of information across SEs while varying the number of lines that serve as connectivity across the SEs. We considered the following sets of lines as overlapping across SEs: 10, 20, 30, 40 and 50% of the DS2 (*n* = 280) (Fig. 1B and Suppl. Figure 2:3). When 10% of the total lines overlapped across the SEs, the remaining 252 lines were divided into six unique sets. Thus, in total 70 lines were used as a calibration set in each SE to predict the genetic value of the prediction set (210 lines) in each SE. For 20, 30, 40 and 50% of the total DS2 lines, 58, 88, 112 and 142, respectively, overlapped across the SEs. The calibration sets for each SE for the four different overlapping sizes were 95, 120, 140 and 165 lines, respectively. The prediction sets in each SE for the four overlapping scenarios were 185, 160, 140 and 115 lines, respectively. Again, the process of line allocation across the SEs was repeated 30 times for each overlapping size scenario.

For all the cross-validation schemes, DS1 and DS3 were used separately to increase the size of the calibration set. The contribution to accuracy of prediction in each SE was then evaluated independently for both DS1 and DS3.

We report two measures of the prediction accuracy: firstly, the Pearson correlation of the predicted GBLUP or PBLUP and the best linear unbiased estimates (BLUE) calculated using all observed records of tested and untested lines in each SE. Secondly, we used a Smith–Hazel (SH) selection index (Smith 1936; Hazel 1943) to define the top 20% of candidates based on GBLUP of the lines estimated from full data and GBLUP (estimated and predicted) of lines in the six SEs obtained from the prediction model. Accuracy was estimated as the proportion of lines that intersected between individuals selected using the GBLUP estimates of the lines from full data and the GBLUP obtained from the prediction model. To calculate the SH index, we used the genetic (from Eqs. 1 and 2) and phenotypic variance–covariance matrices and vector of economic weight (0.25, 0.1, 0.3, 0.2, 0.1, 0.05) that optimized the ability to select promising candidates across the SEs, thus maximizing genetic gain. The latter method was used to reflect the advancement decision strategy in the early yield testing stages in the CIMMYT spring wheat breeding program (Suppl. Figure 1).

To assess the accuracy of GBLUP as a selection decision metric in the early yield testing stages, we estimate the BLUE and GBLUP of the 1260 DS1 lines and the 280 DS2 advanced lines using the full data set. Given that the 1260 DS1 lines were only evaluated in a single SE (B5IR), only information from this SE was used for this analysis. We calculated the Pearson correlation of the GBLUP obtained for the complete 280 DS2 lines as well as BLUEs in the two stages of yield testing. We also measured accuracy as percentage of lines that intersect between select top (10, 20, 30, 40 and 50%) lines in the two testing stages to account for possible selection intensity in the early yield testing stage.

### Genomic selection models

We fitted a multi-environment linear mixed model in ASREML (Gilmour 1999) for the sparse testing aided GP analysis using DS2, combined DS1 and DS2 as well as combined DS2 and DS3 as follows:

1 $${\varvec{y}} = {\mathbf{1}}_{n} \mu + {\varvec{X}}_{1} {\varvec{b}}_{1} + {\varvec{Z}}_{1} {\varvec{u}}_{1} + {\varvec{Z}}_{2} {\varvec{u}}_{2} + {\varvec{Z}}_{3} {\varvec{u}}_{3} + {\varvec{Z}}_{4} {\varvec{u}}_{4} + {\varvec{Z}}_{5} {\varvec{u}}_{{\mathbf{5}}} + {\varvec{\varepsilon}}$$

2 $${\varvec{y}} = {\mathbf{1}}_{n} \mu + {\varvec{X}}_{1} {\varvec{b}}_{1} + {\varvec{Z}}_{1} {\varvec{u}}_{1} + {\varvec{Z}}_{2} {\varvec{u}}_{2} + {\varvec{Z}}_{3} {\varvec{u}}_{3} + {\varvec{Z}}_{4.1} {\varvec{u}}_{4.1} + {\varvec{Z}}_{5.1} {\varvec{u}}_{{{\mathbf{5}}{\mathbf{.1}}}} + {\varvec{\varepsilon}}$$

For single-environment analysis, to evaluate the efficiency of GBLUP as a selection decision metric in the early yield testing stages we fitted a linear mixed model as follows:

3 $${\varvec{y}} = {\mathbf{1}}_{n} \mu + {\varvec{X}}_{1} {\varvec{b}}_{1} + {\varvec{Z}}_{1.1} {\varvec{u}}_{1.1} + {\varvec{Z}}_{2.1} {\varvec{u}}_{2.1} + {\varvec{Z}}_{3} {\varvec{u}}_{3} + {\varvec{Z}}_{4.2} {\varvec{u}}_{4.2} + {\varvec{\varepsilon}}$$

where ***y*** (*n* × 1) is the vector of phenotypes of the lines measured in the environments (1…k), *μ* is the overall mean and ${\mathbf{1}}_{n}$ (*n* × 1) is a of vector ones, ${\varvec{b}}_{1}$ is a fixed effect of replication, ${\varvec{u}}_{1}$ is a random effect of SE, ${\varvec{u}}_{1.1 }$ is the random effect of the genomic effect of *g*-th line, ${\varvec{u}}_{2}$ is the random effect of the interaction between the genomic effect of *g*-th line and *k*-th SE, ${\varvec{u}}_{2.1}$ is the random effect of replication nested within trial, ${\varvec{u}}_{3}$ is the random effect of the trial, ${\varvec{u}}_{4}$ is the random effect of replication nested within SE and trial, ${\varvec{u}}_{4.1}$ is the random effect of replication nested within SE, trial and year for the multi-year dataset, ${\varvec{u}}_{4.2}$ is the random effects of incomplete block nested within replication and trial, ${\varvec{u}}_{5}$ is the random effects of incomplete block nested within replication, trial and SE, ${\varvec{u}}_{5.1}$ is the random effects of incomplete block nested within replication, trial, SE and year for the multi-year dataset. The number of fixed and random effects are represented as n and p, while ${\varvec{X}}_{n}$ and ${\varvec{Z}}_{p}$ are incidence matrices for fixed and random effects, respectively. The variance of the random effects ${\varvec{u}}_{2}$, ${\varvec{u}}_{2.1} , {\varvec{u}}_{3}$, ${\varvec{u}}_{4}$, ${\varvec{u}}_{4.1}$, ${\varvec{u}}_{4.2}$, ${\varvec{u}}_{5}$ and ${\varvec{u}}_{5.1}$ was assumed to be distributed as:

4 $${\varvec{u}}_{{\varvec{p}}} \sim N\left( {{\mathbf{0}}, {\varvec{I}}_{{\varvec{p}}} \sigma_{{{\text{up}}}}^{2} } \right)$$

where ${\varvec{I}}_{{\text{p}}}$ and $\sigma_{up}^{2}$ are the identity matrix and variance of the *p-*th random effect expect ${\varvec{u}}_{2.1}$ where ***I*** is either genomic (***G)*** or pedigree (***A***) relationship matrix. In Eqs. 1 and 2, the random GEI effect ${\varvec{u}}_{2}$ is defined as the Kronecker product ($\otimes$) between ***G*** or ***A*** relationship matrix with dimension *g* × *g* and the k × k variance–covariance matrix of the genomic effect of lines in and between SEs (${\varvec{G}}_{o}$). For combined DS1 and DS2 analysis, the ***G or A*** relationship matrix is a block diagonal matrix such that only 280 lines that overlap across the two datasets were used in the analysis as the remaining 980 lines do not have marker information.

5 $$u_{2} \sim N\left[ {0,\left( {{\varvec{G}} \otimes {\varvec{G}}_{o} } \right)} \right]$$

The covariance of the genomic effect of the line ${\varvec{u}}_{2}$ in multi-environment model can be represented as:

6 $${\text{Cov}}\left( {{\varvec{u}}, {\varvec{u}^{\prime}} = {\varvec{G}}_{o} \otimes {\varvec{G}}} \right)$$

7 $${\varvec{G}}_{o} \otimes {\varvec{G}} \, = \left[ {\begin{array}{*{20}c} {\sigma_{g1}^{2} } & {\sigma_{g12} } & {\begin{array}{*{20}c} \cdots & {\sigma_{g1k} } \\ \end{array} } \\ {\sigma_{g21} } & {\sigma_{g2}^{2} } & {\begin{array}{*{20}c} \cdots & \cdots \\ \end{array} } \\ {\sigma_{gk1} } & {\begin{array}{*{20}c} \vdots \\ \vdots \\ \end{array} } & {\begin{array}{*{20}c} {\begin{array}{*{20}c} \ddots \\ \ldots \\ \end{array} } & {\begin{array}{*{20}c} \vdots \\ {\sigma_{gk}^{2} } \\ \end{array} } \\ \end{array} } \\ \end{array} } \right] \otimes {\varvec{G}}$$

where ${\varvec{G}}_{o}$ ${G}_{\mathrm{o}}$ ${G}_{o}$ represents the *k* × *k* variance–covariance matrix of the genomic effect of lines in the SEs. The diagonal of the ${\varvec{G}}_{o}$ matrix is the additive genetic variance ${\upsigma }_{{{\text{g}}_{{\text{k}}} }}^{2}$ within the *k*-th SE. The off-diagonal (${\sigma }_{{g}_{1k}}$) elements represent the genetic covariance between SEs.

The factor analytic (FA) model which is a parsimonious approach for fitting GEI and complex covariance structure among environments (Piepho 1998; Smith et al. 2001; Crossa et al. 2004; Oakey et al. 2016; Smith and Cullis 2018) was used in this study. We use the extended FA (XFA) model that allows a non-full rank variance matrix for the GEI effects; therefore, the mixed model equation is sparser, resulting in reduced computational requirements compared to the standard FA model, as reported in Thompson et al. (2003) and Meyer (2009). In general, FA identifies one or few factors underlying the correlation among environments by their relationship to unobservable latent variables. Thus, GEI is modeled as an interaction between the genomic effect of the *g*-th line and one or few factors underlying the environmental influences on the line (Piepho 1998; Smith et al. 2001; Crossa et al. 2004; Kelly et al. 2007).

FA model for Cov(${{\varvec{u}}}_{{\varvec{g}}},{{\varvec{u}}}_{{\varvec{g}}}^{\mathbf{^{\prime}}}$) is expressed as:

8 $$({\mathbf{\Lambda \Lambda }}^{\prime} \, + {{\varvec{\Psi}}}) \otimes {\varvec{G}}$$

where **Λ** is a *k* × *m* matrix of loading factors and the columns of **Λ** are associated with the environmental loadings for the *m*-th latent factor. **Ψ** is a *k* × *k* heterogeneous diagonal matrix with specific environment genetic variances $\left({{\varvec{\Psi}}}_{\mathrm{k}}\right)$ on the diagonal and zero covariance between environments.

The residual variance for Eqs. 1, 2 and 3 can be specified as:

9 $$\varepsilon \sim N\left( {0,{\varvec{R}}} \right)$$

where ***R*** is a block diagonal matrix with the error variances within SEs expect for Eq. 3. To allow separate spatial covariance structure within SEs, ***R*** for each SE was defines as:

10 $${\varvec{R}}_{k} = \sigma_{k}^{2} \left[ {{\mathbf{AR1}}\left( {p_{c} } \right) \otimes {\mathbf{AR1}}\left( {p_{r} } \right) } \right]$$

${\sigma }_{\mathrm{k}}^{2}$ is the spatial residual variance in *k*-th SE and $\mathbf{A}\mathbf{R}1\left({p}_{\mathbf{c}}\right)\otimes \mathbf{A}\mathbf{R}1\left({p}_{\mathrm{r}}\right)$ is the Kronecker product of first-order autoregressive processes across columns and rows, respectively.

Plot-level heritability for *k*-th SE was derived from the variance components obtained from the model as:

11 $$h_{k}^{2} = \frac{{\sigma_{gk}^{2} }}{{\sigma_{gk}^{2} + \sigma_{\varepsilon k}^{2} }}$$

where ${\sigma }_{{\mathrm{g}}_{\mathrm{k}}}^{2}$ and ${\sigma }_{{\upvarepsilon }_{\mathrm{k}}}^{2}$ are the genetic, residual variance estimates for *k*-th SE.

Best linear unbiased estimates (BLUEs) for the lines were computed using Eqs. 1–3 while allowing ${{\varvec{u}}}_{2}$ and ${{\varvec{u}}}_{2.1}$ to be fixed instead of random. The BLUEs were used as reference genotypic value to compare the PBLUP or GBLUP models.

## Results

### GBLUP gives higher correlation values compared to BLUEs for stage 1-to-2-line advancement

**Fig. 2:**
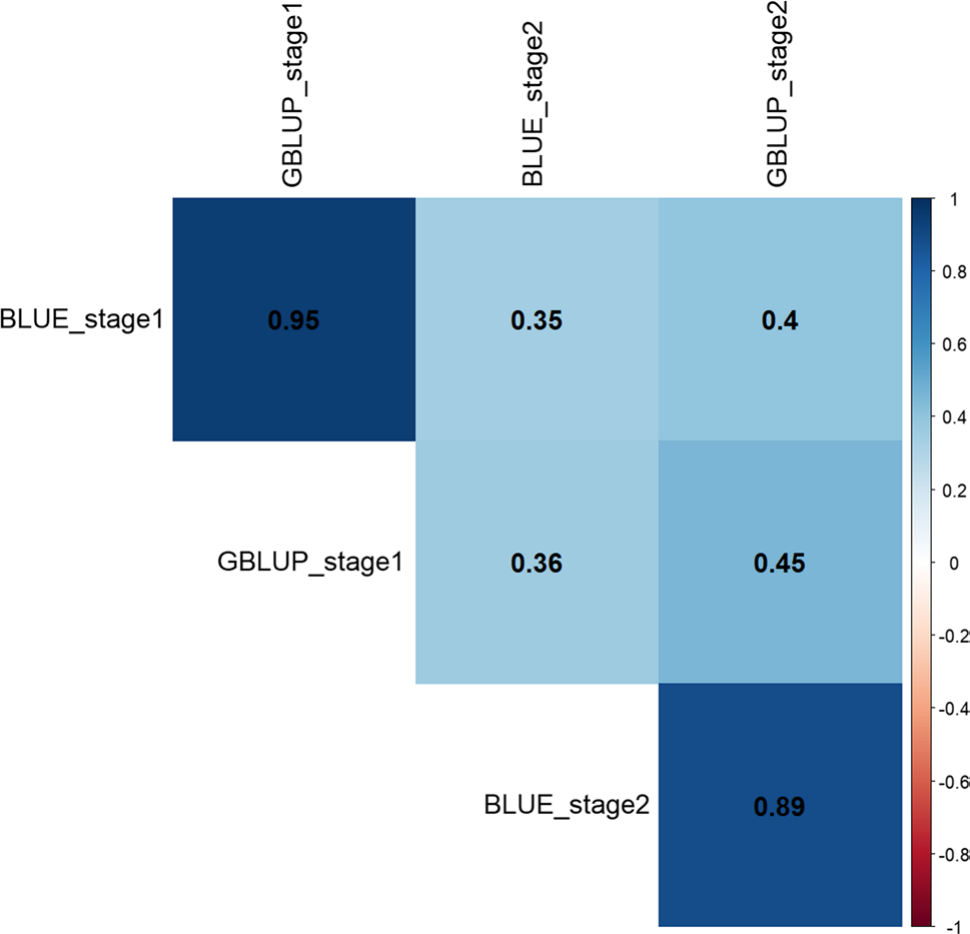
Correlation estimates between the 280 lines selected from 1260 lines in stage 1 and further evaluated in stage 2 using GBLUP and BLUE calculated from complete dataset as the selection criteria. GBLUP_stage 1 and GBLUP_stage 2 denote GBLUP estimates of the same 280 lines in stage 1 using DS1 and stage 2 using DS2, respectively. Similarly, BLUE_stage 1 and BLUE_stage 2 represent BLUE estimates of the lines in stage 1 and stage 2, respectively

As expected, GBLUP was found to be a more effective metric for line advancement from stage 1 (DS1) to stage 2 (DS2). The GBLUP correlation between the two yield testing stages was 0.45 compared to 0.35 when BLUEs were used as the advancement decision metric (Fig. 2). GBLUP consistently improved selection accuracy compared to BLUE as the selection decision metric based on the proportion of lines that overlap between the two testing stages for the select top (10, 20, 30, 40 and 50%) of lines (Suppl. Figure 4).

### Heritability was high within SEs and genetic correlations varied significantly between pairs of SEs

**Fig. 3:**
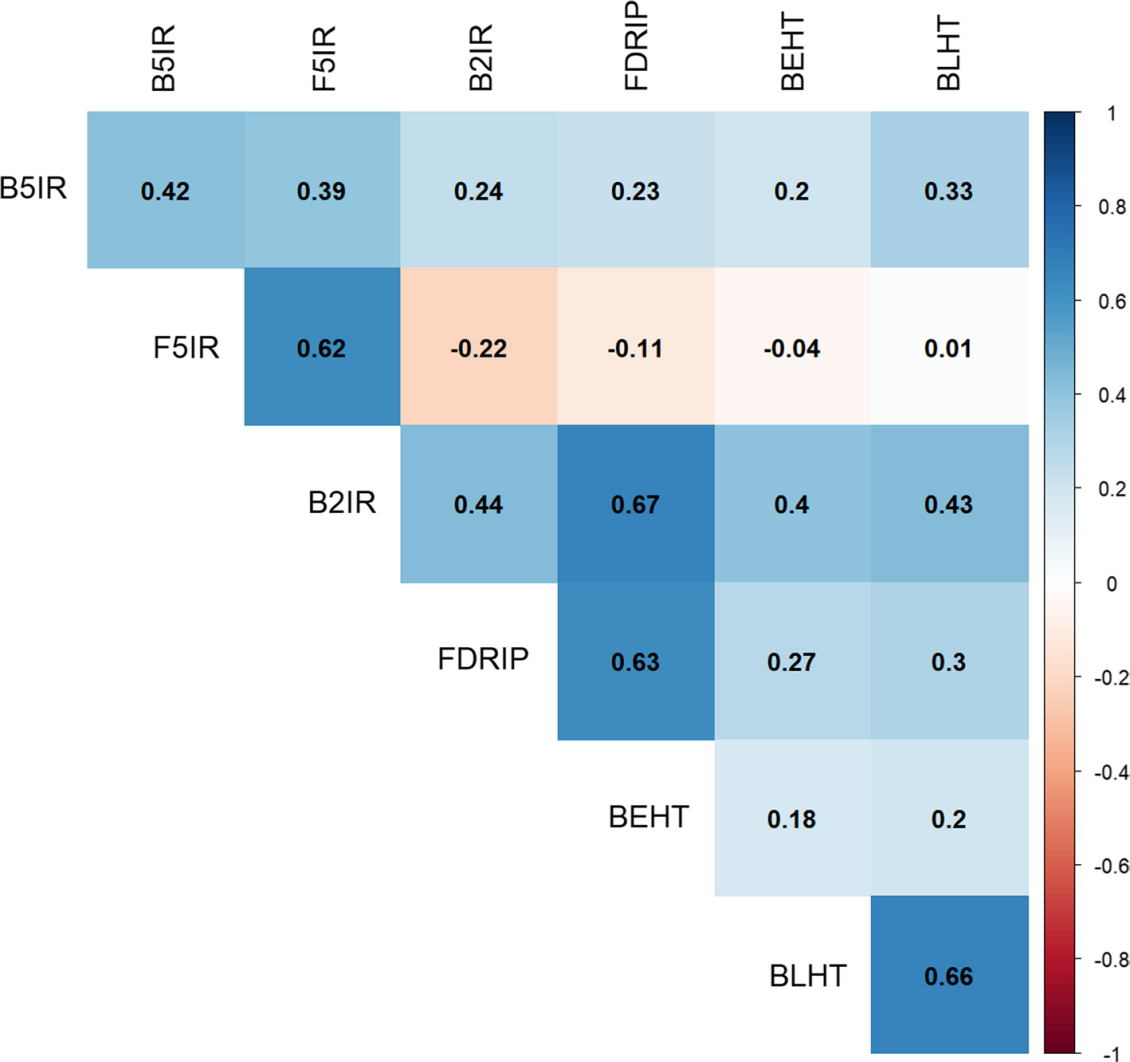
Plot-level heritability (diagonal) and genetic correlation between pairs of SEs (upper diagonal) from factor analytic model analysis of complete DS2

The plot-level heritability for SEs in DS2 (stage 2) ranged from moderate to high except BEHT which had a low plot-level heritability value of 0.18 (Fig. 3). Similar results were obtained in the analyses when DS1 or DS3 were used to augment the calibration set in DS2 (Suppl. Figures 5 and 6). The genetic correlation between SEs in DS2 ranged from − 0.04 to 0.67. Furthermore, when SEs were defined across years (B5IR, F5IR, B2IR, FDRIP, BEHT, BLHT and B5IR*; with B5IR* representing the B5IR SE in DS1 (stage 1)), the genetic correlation between SEs ranged from − 0.05 to 0.85 (Suppl. Figure 5). The genetic correlation between B5IR* and B5IR was high and positive (0.80), whereas that between B5IR* and B2IR, and FDRIP was negative (− 0.11 and − 0.05, respectively). In the analysis where DS3 was used to increase the size of calibration set in DS2, most of the SEs in DS3 denoted by (**) suffix have moderate to high genetic correlation with SEs in DS2 (Suppl. Figure 6).

### Predictive ability increases dependent on heritability, SE correlations and calibration set size using different spare testing strategies

**Fig. 4:**
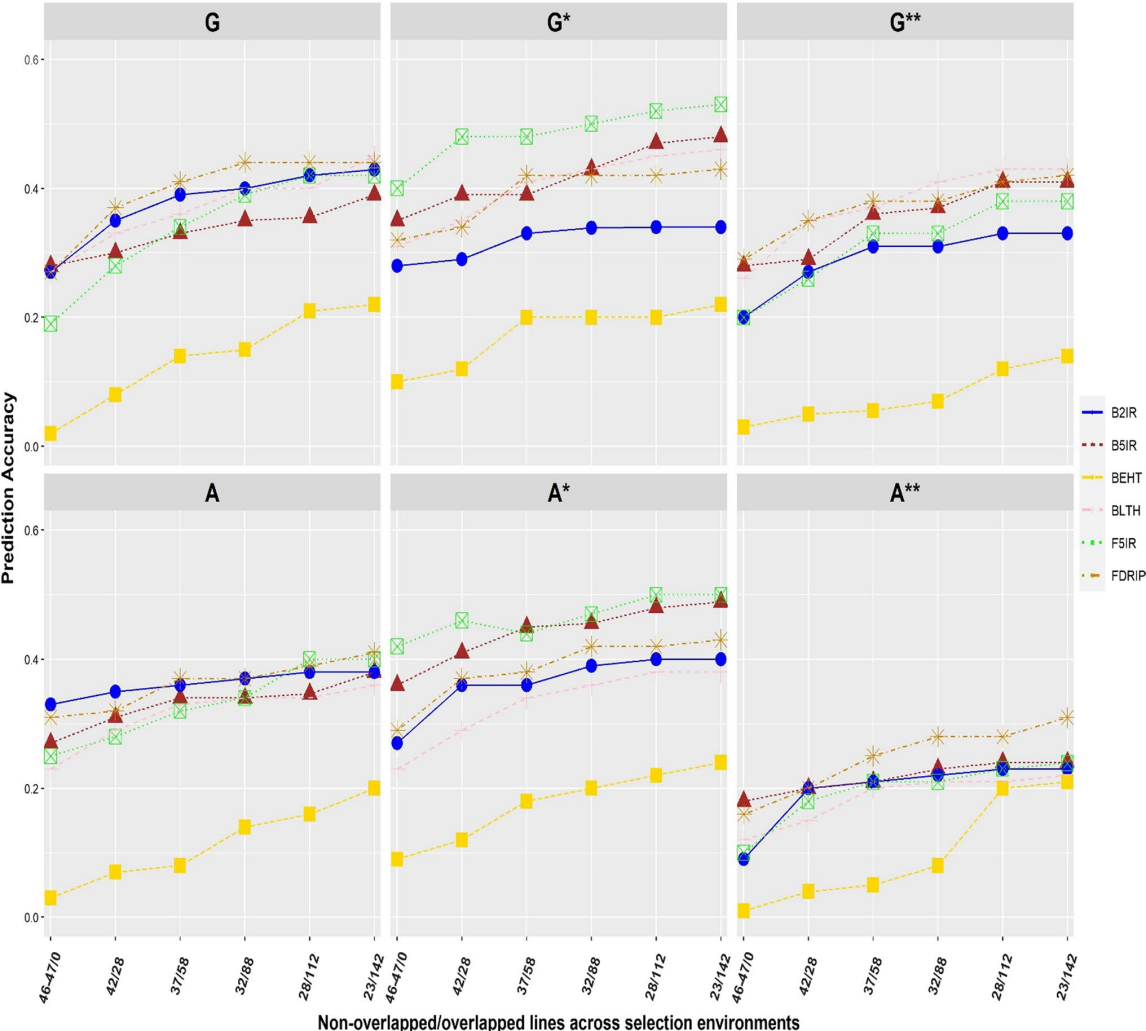
Predictive ability of untested lines in each SE for the different sparse testing strategies. The different colors denote SEs (B2IR, B5IR, BEHT, BLHT, F5IR and FDRIP). G, G* and G** represent predictive ability obtained as the Pearson correlation of the predicted GBLUPs to the observed BLUEs. The suffix (* and **) represents predictive ability obtained when calibration set was augmented with DS1 and DS3, respectively. Also, A, A* and A** represent predictive ability obtained as the Pearson correlation of the predicted PBLUPs to the observed BLUEs with the suffix (* and **) as above

Predictive ability increased with higher heritability and genetic correlation between SEs. The BEHT SE had the lowest plot-level heritability (0.18) and had consistently low predictive ability irrespective of the sparse testing strategy and prediction model used (Fig. 4). Higher predictive ability was generally observed in each SE with increased number of genotypes overlapping across SEs. For instance, using only the DS2 the predictive ability increased by 42, 111, 59, 64, 989 and 67%, respectively, when 50% of the genotypes overlapped across the SEs (B5IR, F5IR, B2IR, FDRIP, BEHT, BLHT) compared to non-overlap of genotypes across the SEs. However, the predictive ability did not increase linearly with expansion in the number of lines that connected the SEs.

The augmentation of the calibration set with DS1 or DS3 improved predictive ability of untested lines for some SEs, especially with DS1 using only the 280 lines evaluated in the B5IR SE. For example, when lines were not repeated across SEs, the predictive ability of the 234 lines in F5IR SE increased by 21 and 1% using DS1 and DS3, respectively, to augment the calibration set. While it increased by 5 and 8% in FDRIP SE using DS1 and DS2, respectively, to increase the size of the calibration set. In general, the use of DS1 to augment the calibration set improved predictive ability in each SE compared to DS3. However, the result of the population structure of the datasets assessed by spectral decomposition of the genomic relationship matrix of the lines in DS2 and DS3 shows significant overlap across the datasets (Suppl. Figure 7). This possibly indicates that genetic correlation between environments influences predictive ability in multi-environment genomic prediction.

The prediction performance of the PBLUP model was similar to the GBLUP model which is not surprising as DS2 consisted of multiple populations with family sizes that ranged from 1 to 9 (Suppl. Figure 8). However, the predictive ability was lowest when DS3 was combined with the calibration set in each SE. For example, with no overlapping lines across SEs, using the DS3 to increase the calibration set size resulted in a predictive ability obtained from the PBLUP model across the SEs ranging from 4 to 31%, while the prediction ability obtained from the GBLUP model ranged from 9 to 46% across the SEs.

### Mimicking selection advancement decision strategy as proxy for prediction performance

**Fig. 5:**
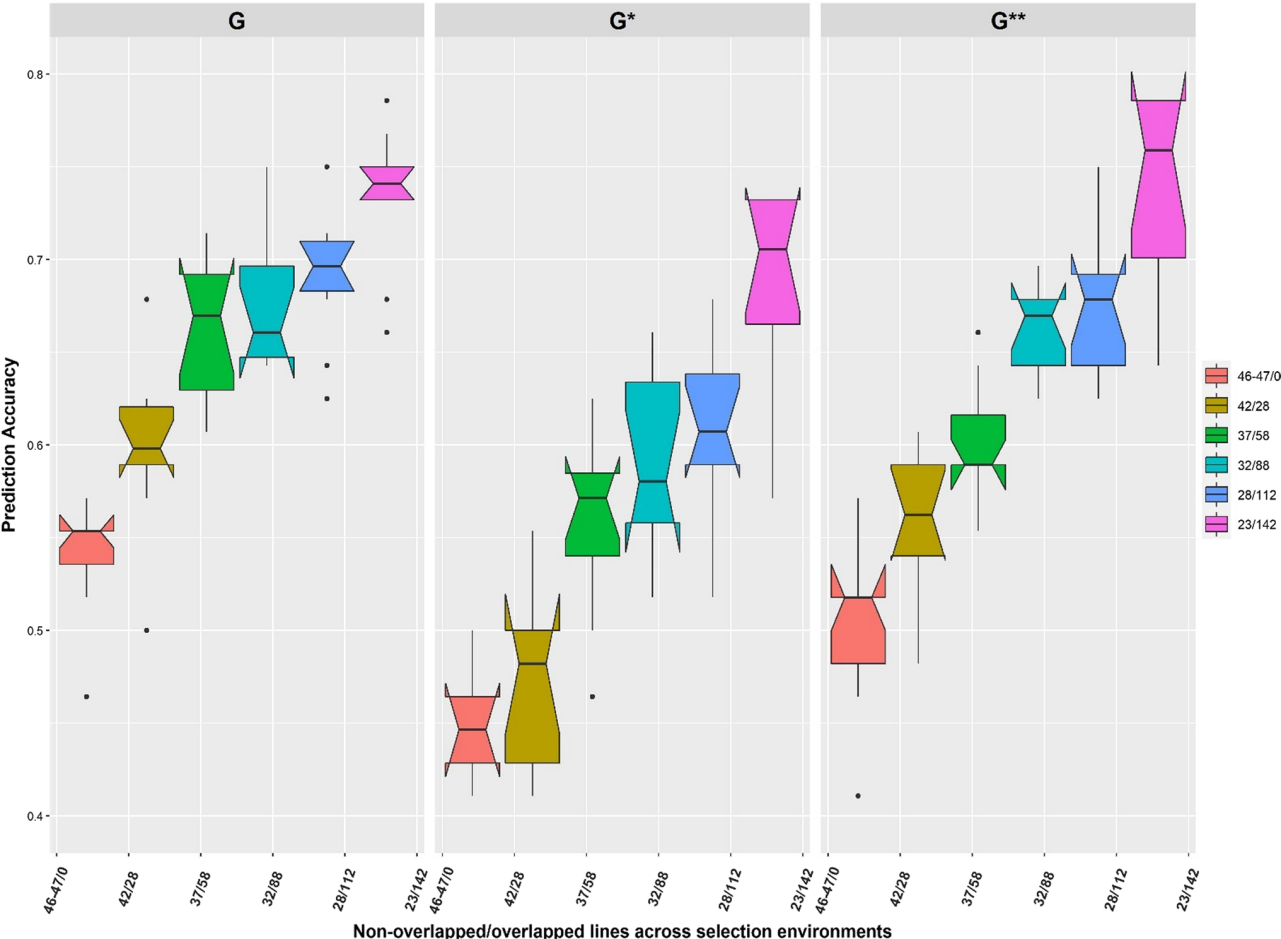
Prediction accuracies obtained for the different allocation of lines to SEs using proportion of lines that intersect between select top 20% lines based on SH selection index model using GBLUP from the prediction model and GBLUP estimates from full data across the SEs. The suffix (* and **) represents prediction accuracy obtained when calibration set was augmented with DS1 and DS3, respectively

The prediction performance improved with increasing size of the calibration set, further corroborating the importance of genetic connectivity across SEs/environments in sparse testing (Fig. 5). Contrary to the prediction performance in each SE, prediction accuracy was higher using DS3 to increase the size of the calibration set compared to DS1 which suggests the relevance of including information from all SEs. In general, the addition of DS1 or DS3 to increase the size of the calibration set did not consistently improve prediction accuracy. This prediction accuracy method reflects the advancement decision strategy in early yield testing stage of the CIMMYT spring wheat breeding program utilizing GBLUP and selection indices to select parent for the next breeding cycle and candidates for further testing.

## Discussion

Genomic prediction is a powerful tool to reduce selection cycle time and increase selection intensity (Meuwissen et al. 2001; Burgueño et al. 2012; Jacobson et al. 2014; Oakey et al. 2016; Crossa et al. 2017; Santantonio et al. 2020). This study aimed to examine sparse testing using GP for increasing first-year yield trial testing across SEs with lines advanced based on GBLUP without increasing breeding costs. Sparse testing using GP in which new lines are evaluated in different but genetically correlated environments relies on utilization of information from closely related individuals within and across environments using multi-environment models (Burgueño et al. 2012; Jarquin et al. 2020; Atanda et al. 2021b).

The multiple populations with small family size ranging in our study from 1 to 9 individuals imply individuals within a family are likely to be absent across environments and this might suggest low predictive ability observed when non-overlapping lines were randomly distributed in different SEs. In this scenario, genetic correlation between pairs of SEs was estimated through replication of alleles across SEs. Generally, increases in genetic connectivity across SEs improved modeling of the genotype by environment interaction. This gave more efficient correlation estimates and consequently better use of information from related lines tested in correlated SEs. Therefore, the improved predictive ability with increased number of lines connecting the SEs suggests that the efficiency of sparse testing aided GP relies on leveraging information within and across environments (Burgueño et al. 2012; Jarquin et al. 2020).

Jarquin et al. (2020) reported improved predictive ability by increasing the number of lines that overlapped across three environments. Due to the data structure, our study did not exclusively investigate factors that might contribute to the proportion of lines that should overlap across the SEs to obtain optimal predictive ability, and this is acknowledged as a key limitation of the study. We hypothesize factors such as population size, the number of crosses per parent, number of half-sib families, yield testing stage and expected predictive ability might largely influence the proportion of lines that overlapped across SEs. Further studies are required to examine the magnitude of each individual factor and their combinations on the fraction of lines that overlap across environments. This will enable optimal/desired predictive ability when implementing sparse testing using GP in breeding.

Given the multiple populations that constitute the datasets used in this study, we suspect differences in QTL-marker linkage phase across the populations might affect the predictive ability. The observed predictive ability might be due to the number of QTLs segregating across the population limiting the influence of QTL-marker linkage phase. Although this assumption was not evaluated here, it was previously reported by Schopp et al. (2017). The authors reported improved predictive ability obtained for half-sib families compared to un-related families due to higher segregation of QTLs among half-sib families rather than consistency of QTL-marker linkage phases across the families. Theoretically, predictive ability improves with increased size of the calibration set. Previous studies (Habier et al. 2007; Clark et al. 2012; Riedelsheimer et al. 2013; Lee et al. 2017; Campos et al. 2013; Atanda et al. 2021a; Lopez-Cruz et al. 2021) have emphasized the influence of size of the calibration set and the influence of degree of relatedness between calibration and prediction set on predictive ability. Considering the degree of genetic variation in the prediction set, there is minimal possibility of improvement by applying genetic optimization criteria accounting for relatedness between prediction set and the individuals in DS3 in our study. This is because it is unlikely to have the same QTL segregating across all the populations in the prediction set. In Atanda et al. (2021a) and Brandariz and Bernardo (2019), historical data were optimized around each bi-parental population. The optimized individuals from historical data were assumed to be in the same QTL-marker LD phase with full-sibs in the prediction population resulting in improved predictive ability. However, in the current study the prediction set comprised several populations with few progenies per cross; therefore, relatedness between individuals in DS3 and the prediction set was ambiguous and did not consistently improve predictive performance.

Unsurprisingly, the predictive performance of the PBLUP model was similar to the GBLUP model. For across population predictions and based on the architecture of the dataset used in this study, the predictive ability depends largely on the variation in genomic and pedigree relationship between families. Therefore, the PBLUP model was able to track segregating QTLs across the families and thus explained a large proportion of the genetic variance among the families. This result agreed with previous studies (Crossa et al. 2010; Albrecht et al. 2011; Schopp et al. 2017; Basnet et al. 2019; Calleja-Rodriguez et al. 2020).

In a breeding program, accurate estimate of selection candidates’ breeding value is critical as a predictor of genetic potential and the ability to generate superior progenies in the subsequent generation (Falconer and Mackay 1996; Gall and Bakar 2002; Zhang et al. 2011; Crossa et al. 2021). In practice, programs are unlikely to have an estimate of true breeding value of genotypes; however, GBLUP can be used as a surrogate of true breeding value. This is especially true for traits with inherent low to medium heritability such as grain yield (Henderson 1975; Gall and Bakar 2002; Zhang et al. 2011; Lell et al. 2021) particularly in the early yield testing stages where genetic merit of lines is evaluated in few environments. According to Lell et al. (2021), the use of GBLUP or BLUE as selection criteria will be influenced by the reliability of the available information (evaluation stage) as well as whether the assumption of genotype independence can be valid in the evaluation stage. As a result, GBLUP superiority over BLUE or BLUE superiority over GBLUP cannot be generalized across evaluation stages. The model underlying BLUE assumes all lines were independent, while GBLUP model incorporates genomic relationship between lines allowing use of the phenotypic information from individual lines and relatives (Henderson 1975; Lell et al. 2021). In the early yield testing stage where genetic merit of lines is evaluated in few environments, our results indicate GBLUP improved selection accuracy compared to BLUE as selection decision metric. Other factors that contribute to phenotypic expression such as environmental variables can also be adjusted for in the GBLUP model to achieve estimation of genetic merit of lines that is potentially close to the true breeding value of the lines (Jarquín et al. 2014; Monteverde et al. 2019; Costa-Neto et al. 2021; Crossa et al. 2021).

## Conclusion

The results from our study show that GBLUP should be used as an advancement decision metric and parental selection criteria in the early yield testing stages, where genetic merit of lines is evaluated in few environments. For programs implementing sparse testing GP for multi-environment yield trials, consideration should be given to the proportion of lines that overlap across environments in the early yield testing stages to increase the size of the SE or selection intensity. Our study suggests including a substantial number of common lines across environments to ensure precise estimation of genetic correlation between environments and to enable improved modeling of GxE interaction effects. In general, sparse testing using GP is a promising strategy for increasing genetic gain in a breeding program by optimizing testing across SE while keeping the breeding costs constant.

## Supplementary Information

Below is the link to the electronic supplementary material.

Supplementary file1 (DOCX 968 KB)

## Data Availability

The dataset used in this study is available in Dataverse at https://hdl.handle.net/11529/10548639.
