# Supplementary material for: Sparse testing using genomic prediction improves selection for breeding targets in elite spring wheat
Source: Theor Appl Genet. 2022 Mar 28;135(6):1939–50. doi: 10.1007/s00122-022-04085-0 (PMC9205816; doi:10.1007/s00122-022-04085-0)
Supplement: Supplementary file 1 — Supplementary file1 (DOCX 968 KB) [file 122_2022_4085_MOESM1_ESM.docx]

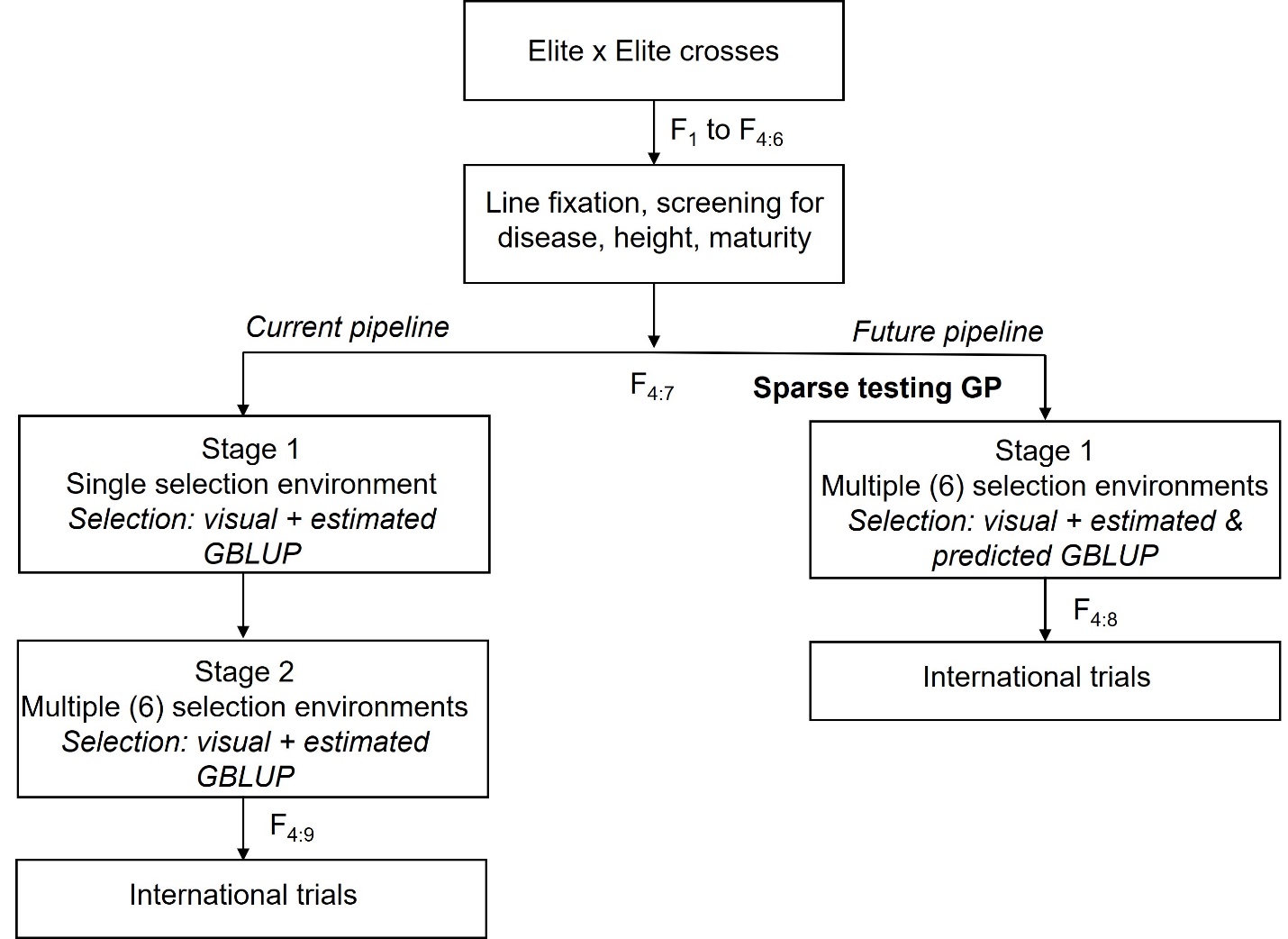


**Supplementary Figure 1: Schematic representation of current and future genomic selection pipeline for the CIMMYT spring wheat breeding program showing the proposed usage of sparse testing using genomic prediction (GP) for Stage 1 testing in multiple selection environments with use of estimated and predicted GBLUP.**


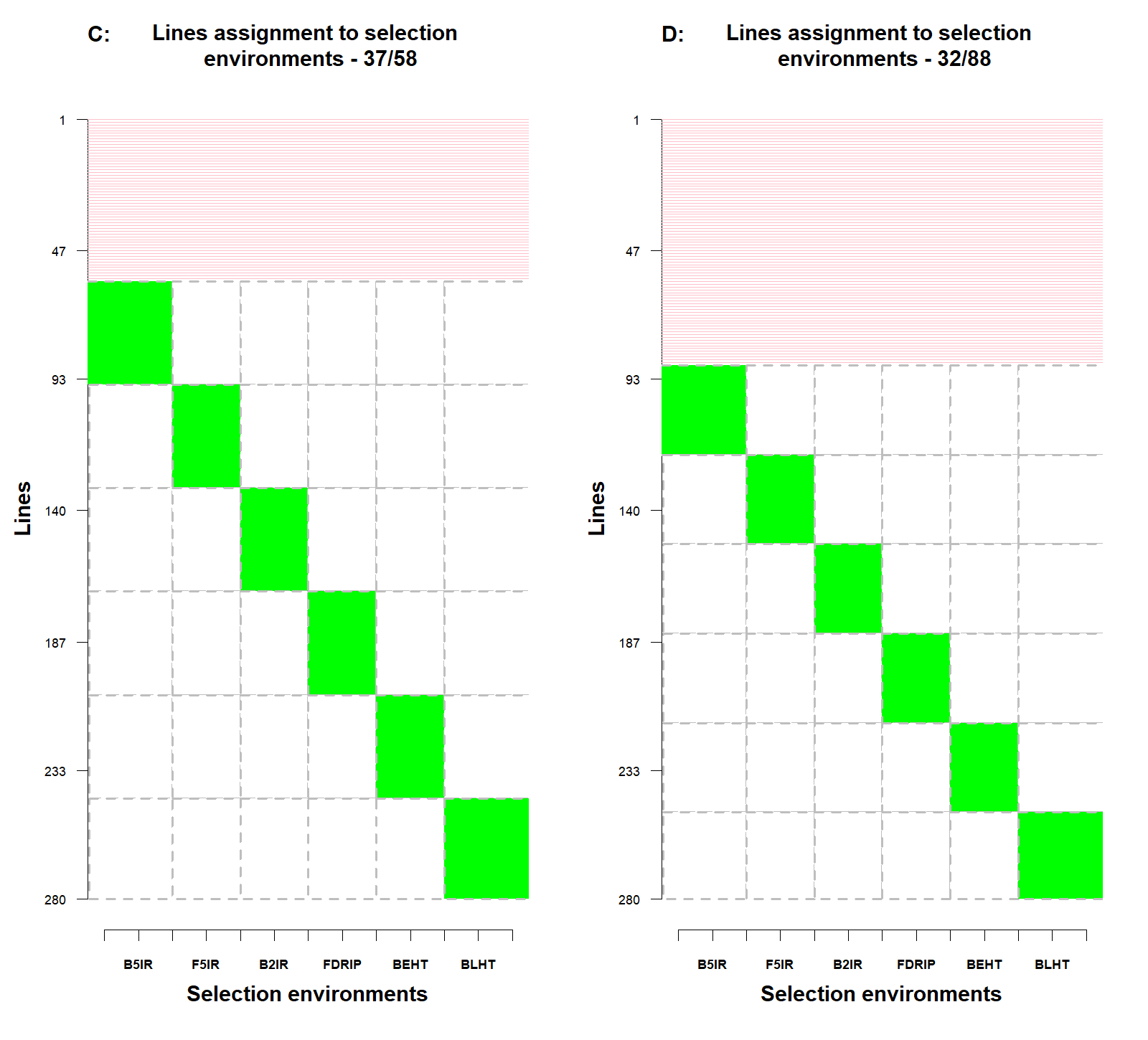


**Supplementary Figure 2: Allocation of 280 lines to the six SEs. Each column represents a discreet SE and the green sections in each column correspond to unique lines tested in SE. C) The green sections correspond to 37 lines unique to each SE while the pink section denotes 58 lines (Approx. 20 percent of 280 lines) common to all SEs. D) The pink section represents 88 lines (Approx. 30 percent of 280 lines) that overlapped across the SEs and the green sections were 32 lines unique to each SE.**


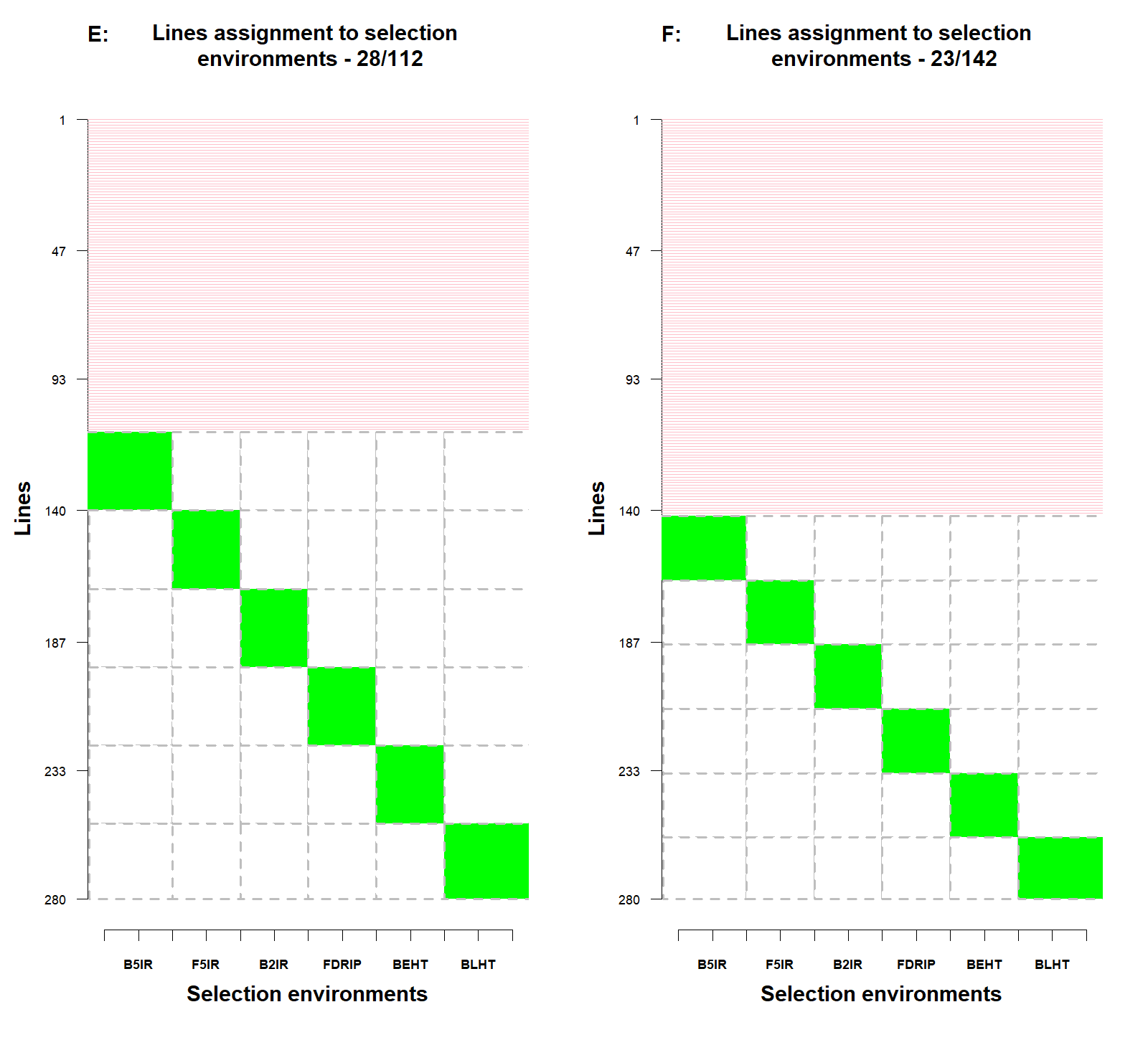


**Supplementary Figure 3: Allocation of 280 lines to the six SEs. Each column represents a discreet SE and the green sections in each column correspond to unique lines tested in each SE. E) The green sections correspond to 28 lines unique to each SE while the pink section denotes 112 lines (Approx. 40 percent of 280 lines) common to all SEs. F) The pink section represents 142 lines (Approx. 50 percent of 280 lines) that overlapped across the SEs and the green sections were 23 lines unique to each SE.**


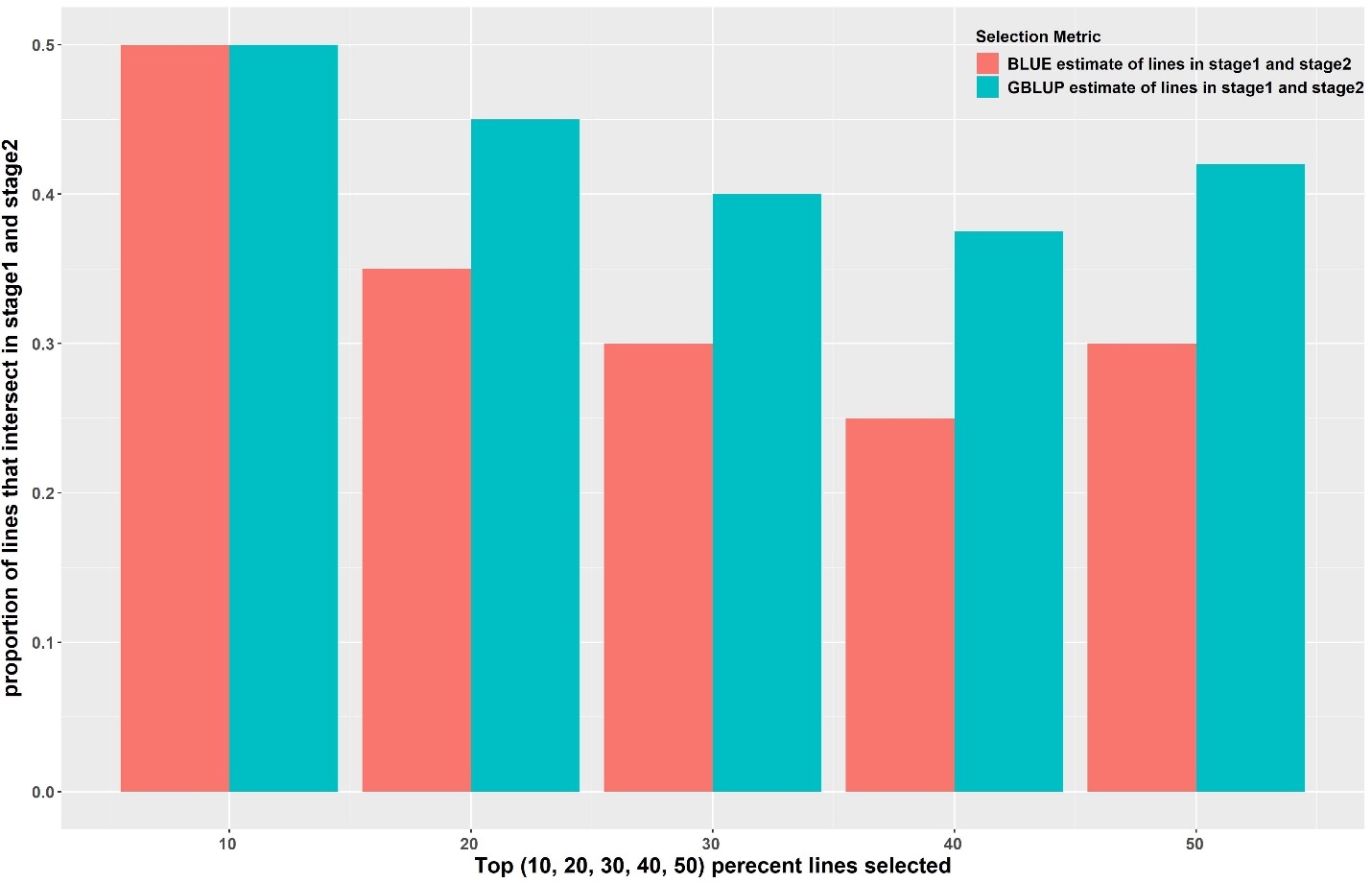


**Supplementary Figure 4: Comparison of accuracy of GBLUP and BLUE as selection advancement decision metric. Accuracy is the proportion of lines for top (10, 20, 30, 40 and 50) selected lines using BLUE and GBLUP as selection criteria that intersect in stage 1 and stage 2 yield testing.**


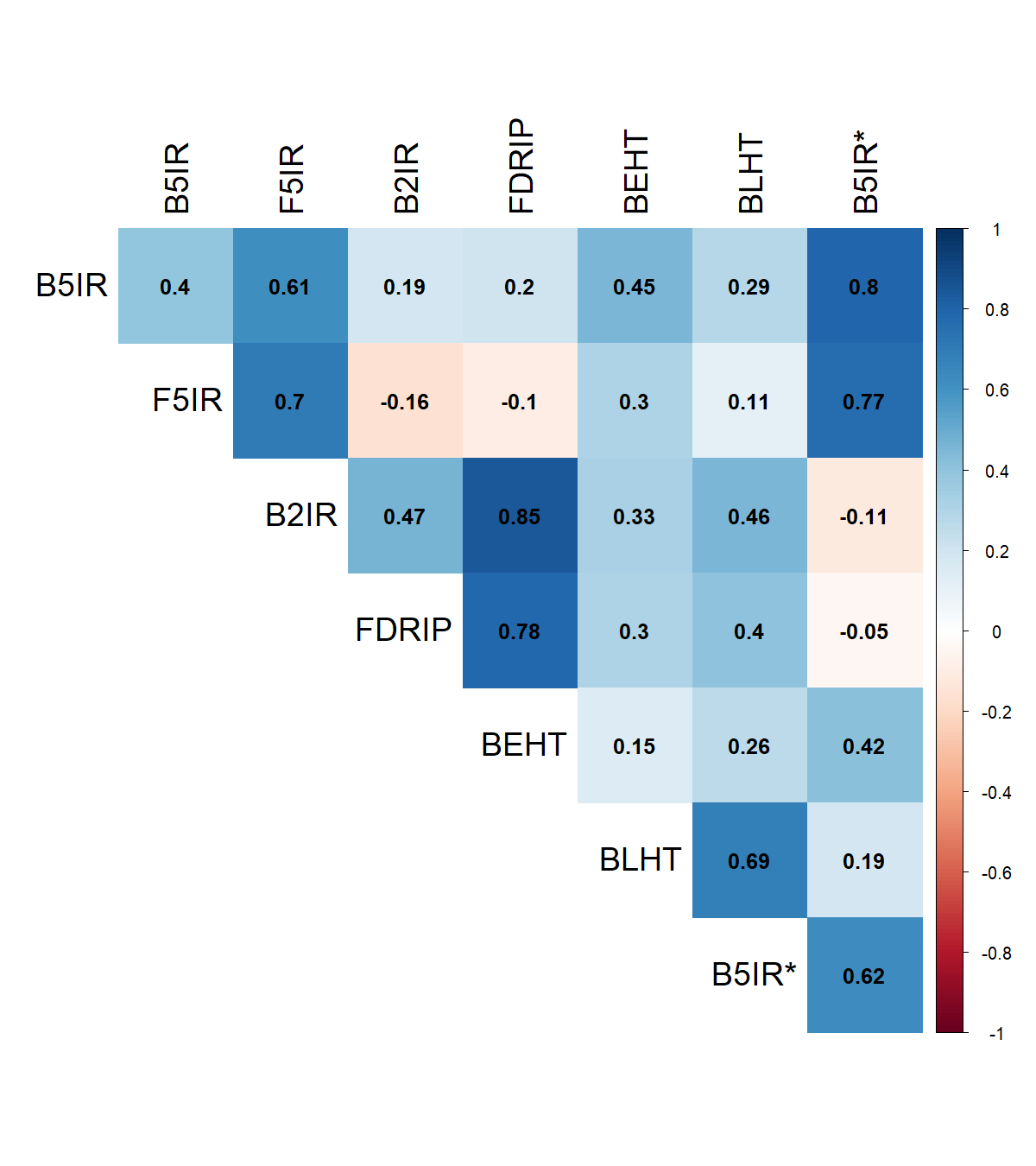


**Supplementary Figure 5: Plot-level heritability (diagonal) and genetic correlation between pairs of environments (upper diagonal) from factor analytic model analysis of complete combined DS1 and DS2. Suffix (*) denotes environment in DS1**


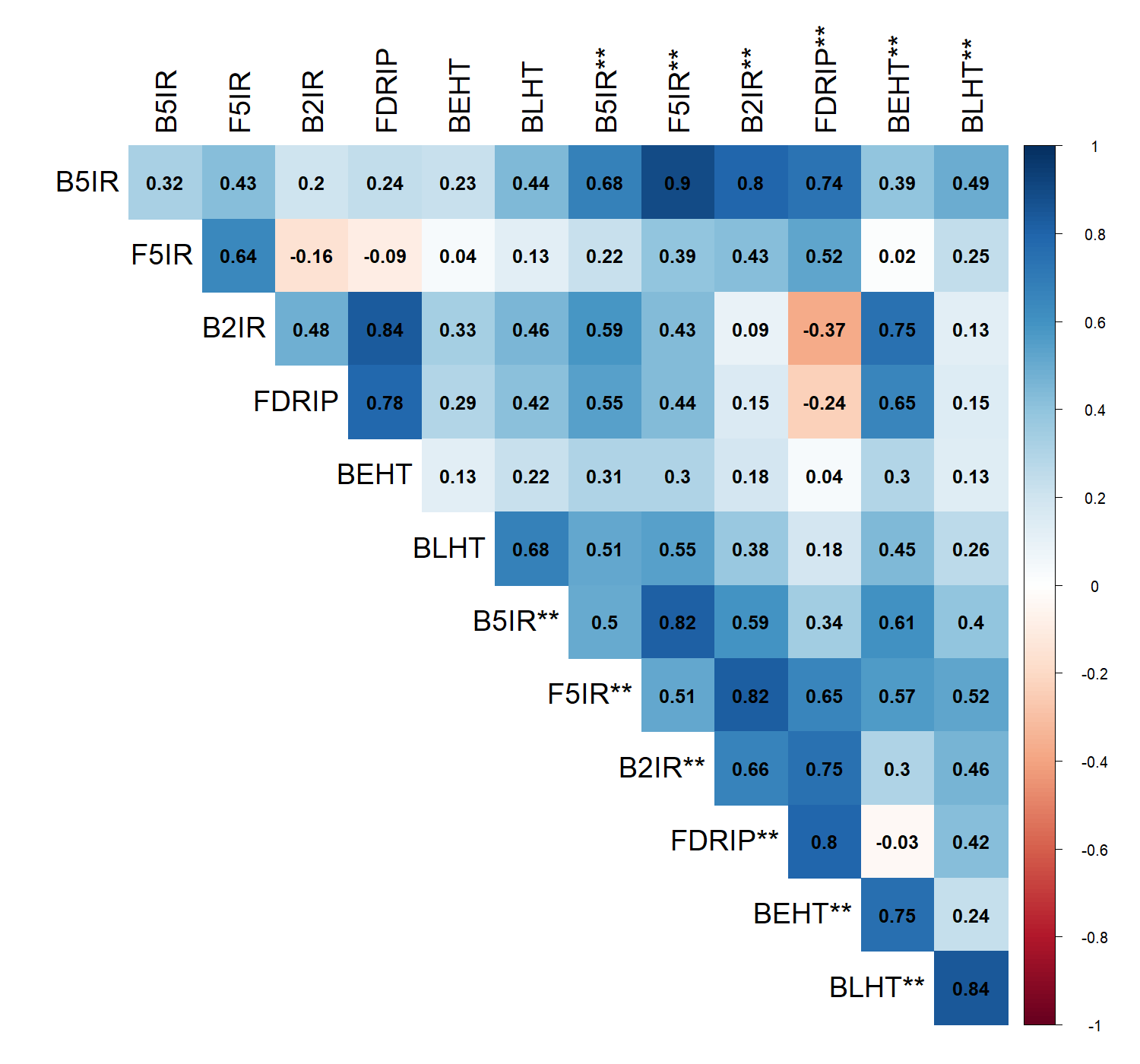


**Supplementary Figure 6: Plot-level heritability (diagonal) and genetic correlation between pairs of environments (upper diagonal) from factor analytic model analysis of complete combined DS2 and DS3. Suffix (**) denotes environment in DS3.**


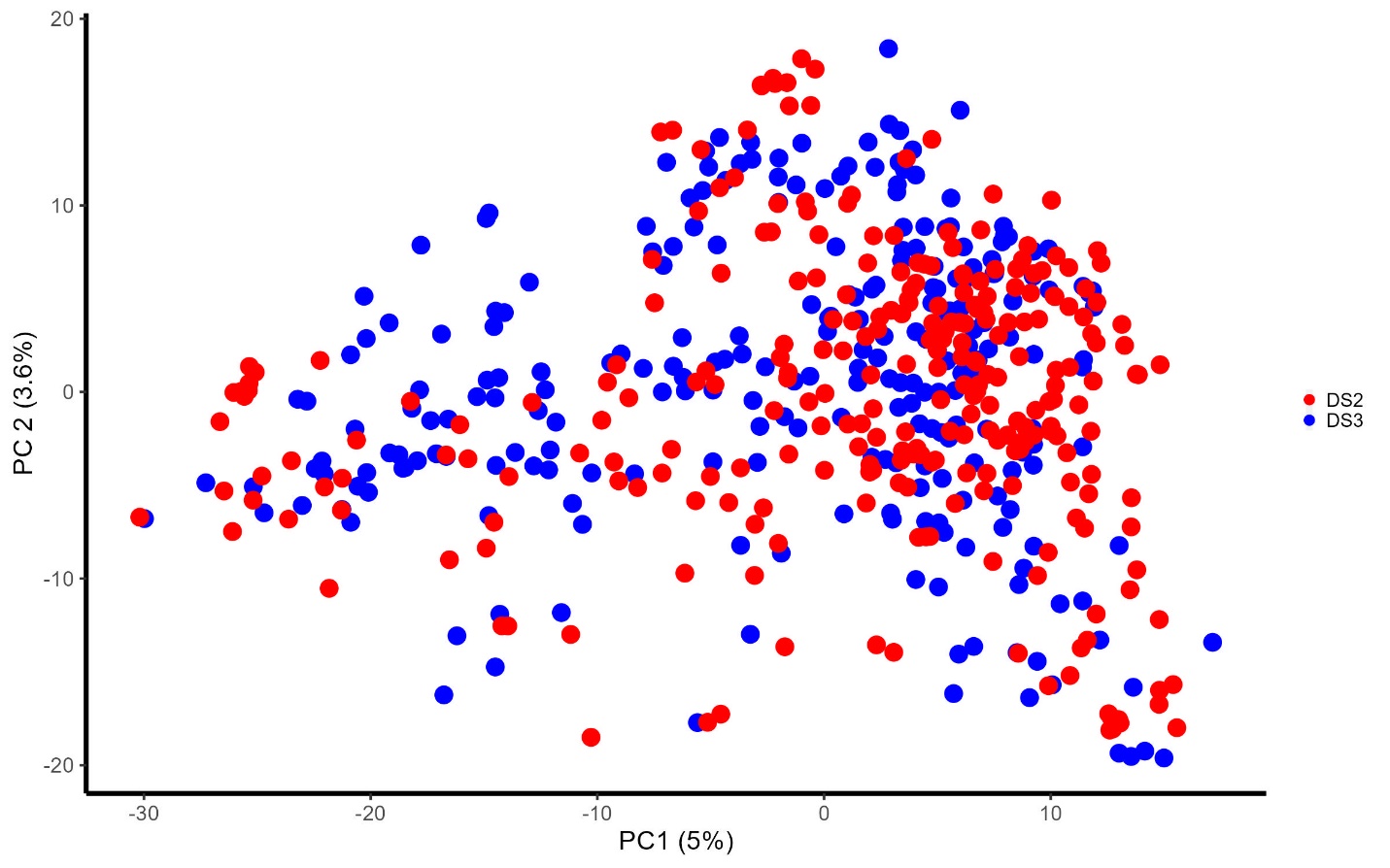


**Supplementary Figure 7: Spectral decomposition of the genomic relationship matrix of the combined D2 (280 lines) and DS3 (252 lines). The plot of the first two principal components shows the diversity within each dataset and interconnectedness across the datasets. Each blue and red dot represent lines in DS2 and DS3 respectively.**


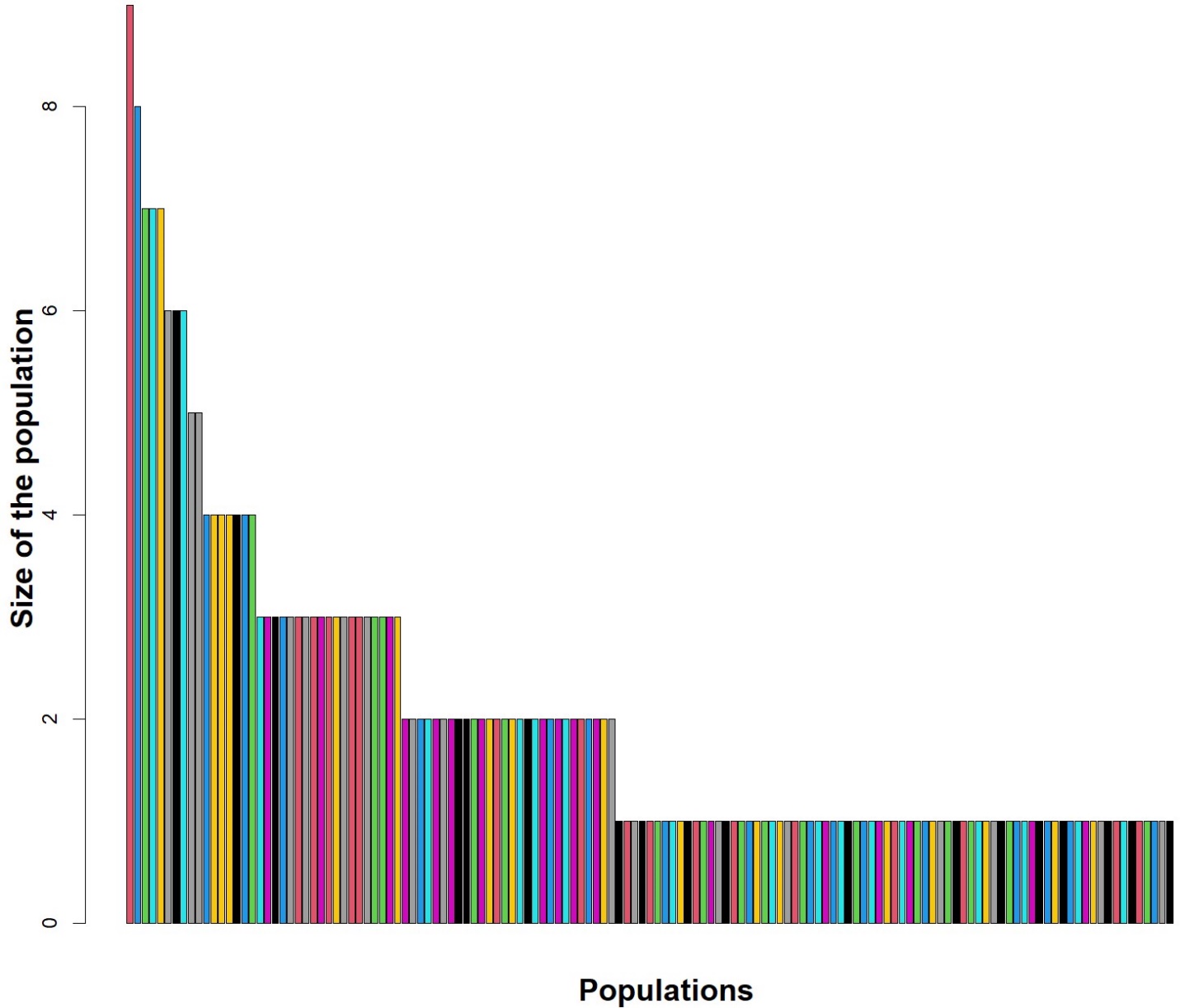
**Supplementary Figure 8: The size of 37 populations in DS2. Each bar represents a population and the y-axis indicate the size of each population.**
